# Epiblepharon in a Case of Anterior Megalophthalmos: A Diagnostic Red Herring

**DOI:** 10.7759/cureus.14304

**Published:** 2021-04-05

**Authors:** Akshay G Nair, Prachi M Agashe, Ashish Doshi

**Affiliations:** 1 Ophthalmic Plastic Surgery and Ocular Oncology, Advanced Eye Hospital and Institute, Navi Mumbai, IND; 2 Pediatric Ophthalmology, Advanced Eye Hospital and Institute, Navi Mumbai, IND; 3 Pediatric Ophthalmology, Horizon Eye Clinic, Mumbai, IND

**Keywords:** congenital glaucoma, buphthalmos, entropion

## Abstract

Epiblepharon is a condition characterized by the presence of a congenital horizontal fold of skin near the upper or lower eyelid margin and rarely requires intervention. In this communication, we present the case of a five-month-old child who had enlarged eyes, tearing, and intense photophobia; and was referred to as a case of congenital glaucoma. Congenital or infantile glaucoma can, indeed present with enlarged eyes, watering, and photophobia. However, in the absence of optic disc cupping and elevated intraocular pressures, a diagnosis of anterior megalophthalmos should be considered, especially in the presence of a very deep anterior chamber. Subsequent evaluation in our case established the diagnosis of anterior megalophthalmos along with concomitant bilateral epiblepharon. The child underwent surgery to correct the epiblepharon, following which, the tearing and photophobia resolved. The clinical characteristics of anterior megalophthalmos and the causality between an enlarged globe and epiblepharon are discussed in this article.

## Introduction

The triad of enlarged eyes, watering, and photophobia in an infant is most often attributed to congenital glaucoma. [1] However, other benign conditions should also be borne in mind when suspecting congenital glaucoma; such as conjunctivitis, corneal injury, in-turning eyelashes from associated conditions such as epiblepharon - a congenital anomaly in which a fold of skin lies across the lower lid margin. [2] Anterior megalophthalmos is also one such condition that typically presents with features similar to those as congenital glaucoma such as megalocornea, very deep anterior chamber depth, and secondary effects of iridodonesis. [3] Anterior megalophthalmos with concomitant epiblepharon has not been reported previously in the literature. Here, we discuss the diagnostic criteria for anterior megalophthalmos and explain the management of this case. 

## Case presentation

A five-month-old Indian boy presented to us with large eyes, photophobia, and tearing noticed since one month of age. The child’s birth and developmental histories were unremarkable. The child was suspected to have congenital glaucoma and was referred for glaucoma surgery. Although the child was photophobic; he was able to maintain steady, central fixation in both eyes. The lower eyelid lashes were found to be turned inwards with some excess skin appearing as a fold on both lower lids. The in-turned lashes were rubbing on the inferior part of the cornea in both eyes. No other systemic abnormalities were found in the child and a pedigree analysis showed a non-consanguineous marriage; the child had no other siblings. Family history was otherwise, non-contributory. 

**Figure 1:**
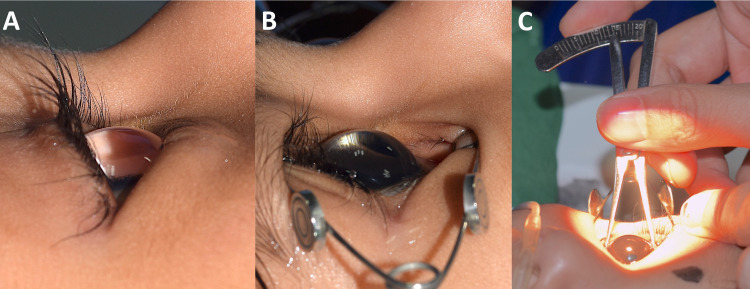
External photograph of the right eye. The fold of skin on the lower lid is visible with no visible lashes. This epiblepharon has led to the lashes being turned in towards the globe. (Figure 1A). The lashes are visible on placing a speculum (Figure 1B). Also visible is the extremely deep anterior chamber. Figure 1C shows the corneal diameter being measured at 14 mm.

**Figure 2:**
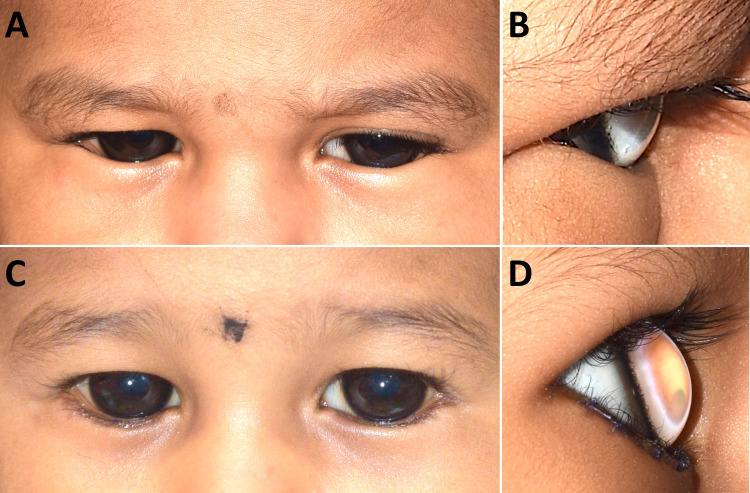
Pre-operative and post-operative comparative photographs. External photograph showing the child to be extremely photophobic pre-operatively. Note the enlarged corneas and the epiblepharon (Figures 2A, 2B). Post-operatively, the spastic tone of the eyelids has reduced with a wider palpebral fissure. Lower lid scars are visible and the epiblepharon is corrected (Figures 2C, 2D).

On examination under anesthesia, the corneas were clear, but on fluorescein staining, superficial punctate erosions were observed on the inferior half of both corneas. In both eyes, a skin fold overlapping the lower lid margin was seen, confirming the diagnosis of epiblepharon (Figure 1A). The anterior chamber was deep (Figure 1B), the lens was clear and the optic disc showed a cup disc ratio of 0.1:1 with a healthy neuroretinal rim and healthy macula in both eyes. The corneal diameters measured 14 mm horizontally and vertically in both eyes (Figure 1C). The biometric measurements were obtained through an immersion A-scan (Table 1). Cycloplegic retinoscopy indicated a refractive error of +1.00 D in each eye and intraocular pressures were 8 mm Hg in both eyes. On performing gonioscopy, the angles were found to be wide open with an enlarged ciliary ring. Based on these findings, the child was diagnosed to have anterior megalophthalmos along with epiblepharon (Figures 2A and 2B).

The child was treated with hourly instillation of carboxymethylcellulose sodium lubricant eye drops 0.5% w/v which improved the photophobia. Observation was advised at this time, in order to wait for spontaneous resolution of epiblepharon. However, this did not happen and at 18 months of age, the child underwent an eyelid surgery that involved resection of a strip of skin and orbicularis muscle along with anchoring of the lower lid retractors to the lower edge of the tarsus. This corrected the epiblepharon which in turn resulted in resolution of watering and photophobia (Figures 2C and 2D).

**Table 1:** Ocular parameters. Table depicting the measured ocular parameters, as measured under general anesthesia.

Parameters	Right Eye	Left Eye
Corneal diameter	14 mm	14 mm
Axial length*	22.56 mm	22.7 mm
Anterior chamber depth*	6.6 mm	6.82 mm
Lens thickness*	3.6 mm	3.8 mm
Vitreous length*	12.42 mm	12.39 mm
Vitreous index (Axial length/Vitreous length x 100)	55%	54%
* Using immersion A-scan		

## Discussion

Anterior megalophthalmos is an uncommon, complex, congenital disorder with stationary enlargement of the anterior segment of the eye [4]. The typical characteristics include clear enlarged cornea (megalocornea) with a horizontal corneal diameter of 13 mm or greater, deep anterior chamber resulting in shortened vitreous length, enlarged ciliary body ring, and the absence of features suggestive of congenital glaucoma [4,5]. It commonly occurs in males; an X-linked inheritance has been reported in 90% of the cases though autosomal recessive and sporadic inheritance have also been documented [6]. The proposed etiology is keratodysgenesis and/or iridogoniodysgenesis; specifically - failure of fusion of the optic cup in the embryonal stages that leads to excessive growth of the nascent cornea. In such cases, the vitreous index is a parameter that helps in the diagnosis of anterior megalophthalmos. The vitreous index (vitreous length / axial length × 100) is noted to be below 69% in patients with anterior megalophthalmos [7]. In our case, the vitreous index was 55% and 54% in the right and left eye, respectively.

The closest differential diagnosis of this condition is infantile glaucoma which could be ruled out clinically based on the findings described. It must be noted that glaucoma has been reported in anterior megalophthalmos - but those are largely secondary to lens subluxation or ciliary body abnormalities. Other reported ocular associations include iris hypoplasia, zonular weakness, angle abnormalities, Descemet’s detachment, posterior dislocation of lens, vitreous degeneration, and retinal detachment [8,9]. Cataract surgery in adults with anterior megalophthalmos has also been reported adequately in the literature [10]. Posterior dislocation of the crystalline lens with retinal detachment has been reported in seven eyes and five eyes, respectively, in a case series describing the ocular abnormalities in anterior megalophthalmos in four children between the age of 4-10 years [5].

Lid abnormalities like epiblepharon have not been reported in association with megalophthalmos. Epiblepharon is an eyelid anomaly commonly found in East-Asian children; a skin fold overlaps the lid margin resulting in cilia against the cornea, corneal irritation, and possibly astigmatism as well. [11] The enlarged cornea and the anterior curvature of the eyeball can lead to the development of epiblepharon. The hypothesis explaining the causality is that the enlarged globe pushes the lower lid downward and alters the balance of forces between the anterior and posterior lamella. This imbalance between the protractors and the retractors of the eyelid, early in childhood, might lead to malalignment of the eyelid, resulting in epiblepharon. This hypothesis is validated by studies that have shown a higher prevalence of lower lid epiblepharon with congenital glaucoma as compared to age-matched controls [12]. Epiblepharon can further cause keratopathy which can then lead to increased blinking and possibly establish a spastic component as well [13]. In our case, the photophobia and tearing, which was due to epiblepharon, was a red herring that could have possibly led to the erroneous diagnosis of infantile glaucoma.

## Conclusions

In summary, anterior megalophthalmos must be considered as a differential diagnosis while assessing any infant who presents with enlarged eyes. Given the structural changes of the anterior segment observed in anterior megalophthalmos, ophthalmologists must be aware that resultant lower lid abnormalities could also be seen. Lid abnormalities due to enlarged globes should be carefully assessed and addressed in order to alleviate the symptoms of tearing and photophobia.
